# Vaccinia Virus Protein A49 Is an Unexpected Member of the B-cell Lymphoma (Bcl)-2 Protein Family*[Fn FN2]

**DOI:** 10.1074/jbc.M114.624650

**Published:** 2015-01-20

**Authors:** Sarah Neidel, Carlos Maluquer de Motes, Daniel S. Mansur, Pavla Strnadova, Geoffrey L. Smith, Stephen C. Graham

**Affiliations:** From the ‡ Department of Pathology, University of Cambridge, Tennis Court Road, Cambridge CB2 1QP, United Kingdom and; the § Department of Microbiology, Immunology, and Parasitology, Universidade Federal de Santa Catarina, Florianopolis, 88040-900 Brazil

**Keywords:** B-cell Lymphoma 2 (Bcl-2) Family, Innate Immunity, NF-κB (NF-KB), Poxvirus, Protein Structure, Viral Protein, Structure-based Phylogenetics, Vaccinia Virus

## Abstract

Vaccinia virus (VACV) encodes several proteins that inhibit activation of the proinflammatory transcription factor nuclear factor κB (NF-κB). VACV protein A49 prevents translocation of NF-κB to the nucleus by sequestering cellular β-TrCP, a protein required for the degradation of the inhibitor of κB. A49 does not share overall sequence similarity with any protein of known structure or function. We solved the crystal structure of A49 from VACV Western Reserve to 1.8 Å resolution and showed, surprisingly, that A49 has the same three-dimensional fold as Bcl-2 family proteins despite lacking identifiable sequence similarity. Whereas Bcl-2 family members characteristically modulate cellular apoptosis, A49 lacks a surface groove suitable for binding BH3 peptides and does not bind proapoptotic Bcl-2 family proteins Bax or Bak. The N-terminal 17 residues of A49 do not adopt a single well ordered conformation, consistent with their proposed role in binding β-TrCP. Whereas pairs of A49 molecules interact symmetrically via a large hydrophobic surface *in crystallo*, A49 does not dimerize in solution or in cells, and we propose that this hydrophobic interaction surface may mediate binding to a yet undefined cellular partner. A49 represents the eleventh VACV Bcl-2 family protein and, despite these proteins sharing very low sequence identity, structure-based phylogenetic analysis shows that all poxvirus Bcl-2 proteins are structurally more similar to each other than they are to any cellular or herpesvirus Bcl-2 proteins. This is consistent with duplication and diversification of a single *BCL2* family gene acquired by an ancestral poxvirus.

## Teaser

**Background:** Poxviruses contain several virulence factors with no identifiable cellular homologues.

**Results:** Vaccinia virus immunomodulatory protein A49 adopts the same fold as cellular and viral Bcl-2 family proteins but does not share their function.

**Conclusion:** Vaccinia virus Bcl-2-like proteins resemble each other structurally but have divergent functions.

**Significance:** Viruses hijack protein scaffolds and evolve them to subvert host-cell immunity.

## Introduction

Vaccinia virus (VACV),^3^ the vaccine used to eradicate smallpox, has a large dsDNA genome encoding ∼200 ORFs (1). About half of these genes are not essential for virus replication but rather affect the virulence and host range of the virus by counteracting the host immune response to infection (2). Understanding how VACV modulates the host immune response can yield unexpected insights into cellular innate immunity (3) and is essential to fully exploit the promise of VACV as a safe vaccine vector (4).

VACV A49 is a virulence factor that inhibits the activation of the host transcription factor NF-κB (5). NF-κB activity is stimulated by extrinsic proinflammatory signals, such as interleukin 1 or tumor necrosis factor α, and by intracellular signals, such as the recognition of foreign cytoplasmic RNA by retinoic acid-inducible gene I-like helicases (6). NF-κB is usually retained, inactive, in the cytoplasm via an interaction with the inhibitor of κB (IκBα). Upon proinflammatory stimulation, IκBα is phosphorylated by IκB kinases and subsequently ubiquitinated and degraded. This allows NF-κB to translocate to the nucleus and promote transcription of cytokines and chemokines, including interferon β, that promote inflammation and an anti-viral state in surrounding cells (7). A49 inhibits the ubiquitination of IκBα by binding β-TrCP, a component of the Skp1·Cullin1·F-box protein (SCF)^β-TrCP^ ubiquitin E3 ligase complex. Mutational analysis suggested that it does so via an N-terminal peptide that binds the β-propeller domain of β-TrCP in an extended fashion, similar to the binding mode observed for β-catenin (8), and thereby blocks binding of IκBα to the same site (5). The importance of inhibiting NF-κB activation to the virus is underscored by the fact that VACV has at least 10 proteins that interrupt this pathway and deletion of any of these genes attenuates the virus (9). Inhibiting the degradation of IκBα appears to be of particular importance for poxvirus virulence; ectromelia virus lacks an A49 homologue but it contains EVM005, an ankyrin family protein that contains an F-box domain and is thought to compete with the F-box region of β-TrCP for binding to Skp1, thus preventing IκBα ubiquitination and degradation (10).

Previous studies have identified that many poxvirus immunomodulatory proteins, including 10 VACV proteins, share structural similarity with the Bcl-2 (B-cell lymphoma 2) family of cellular proteins despite lacking identifiable sequence similarity (11–16). Cellular members of the Bcl-2 family generally share four Bcl-2 homology domains (BH1–BH4) in their primary sequence, and they are key regulators of apoptosis (17). Whereas poxvirus Bcl-2-like proteins F1, M11, and N1 all act to inhibit apoptosis, other poxvirus Bcl-2 family proteins have instead evolved to inhibit activation of the transcription factors NF-κB or IRF3 (9, 13). Of particular interest is VACV protein N1, which inhibits both apoptosis and NF-κB via two distinct molecular surfaces; inhibition of apoptosis is mediated via a hydrophobic surface groove, whereas inhibition of NF-κB is disrupted by mutating a surface on the opposite face of the protein that mediates N1 dimerization both in solution and in cells (18).

Homologues of A49 are found only in a subset of orthopoxviruses (5). Aside from a stretch of six amino acids at its N terminus, the 18.8-kDa A49 protein and its orthologues in other orthopoxviruses share no identifiable sequence identity with any protein of known structure or function. To further investigate the function of A49, we solved its structure to 1.8 Å resolution by x-ray crystallography. Unexpectedly, the structure showed that A49 possesses a Bcl-2-like fold despite not sharing sequence identity with known cellular or poxvirus Bcl-2 family proteins. Structure-based phylogenetic analysis shows that A49 is most closely related to other poxvirus Bcl-2-like proteins, consistent with its evolution from an ancestral poxvirus *BCL2* family gene by means of gene duplication and diversification.

## EXPERIMENTAL PROCEDURES

### 

#### 

##### Expression Vectors

For bacterial expression, *A49R* from VACV Western Reserve (WR) was amplified using KOD HiFi DNA polymerase (Novagen) with forward primer 5′-AGGAGATATACCATGGATGAAGCATATTACTCTGGCAAC-3′ and reverse primer 5′-GTGATGGTGATGTTTCAAATATCGTTCGCGGATATCATTAG-3′ and cloned into pOPINE (19), adding a C-terminal LysHis_6_ fusion tag (full-length A49). A truncated A49 construct lacking residues 1–12 (A49 Δ12) was cloned into pOPTnH, a pOPT (20) vector modified to encode a C-terminal LysHis_6_ tag, following amplification using Platinum TaqDNA polymerase high fidelity (Invitrogen) with forward primer 5′-GGAAGTCATATGGTACTCGGATACGTGTCCGATATGCATAC-3′ and reverse primer 5′-GGAAGTGGATCCCAAATATCGTTCGCGGATATCATTAGACAATTG-3′ containing NdeI and BamHI restriction sites (underlined), respectively. C-terminally His_6_-tagged N1 in pET24a was described previously (21).

For mammalian expression, VACV WR *A49R* was amplified using KOD HiFi DNA polymerase (Novagen) with forward primer 5′-AAGTTCTGTTTCAGGGCCCGGATGAAGCATATTACTCTGGCAAC-3′ and reverse primer 5′-ATGGTCTAGAAAGCTTTACAAATATCGTTCGCGGATATCATTAG-3′. The PCR product was cloned into pOPINF (19), adding an N-terminal His_6_ tag and rhinovirus 3C protease site (nHis-A49). nTAP-A49, Myc- and TAP-β-TrCP (5), FLAG-B14 (22), FLAG-M11 (13), and HA-Bak and HA-Bax (12) have all been described.

##### Protein Production and Characterization

N1 was expressed and purified as described previously (12). Full-length A49 and A49 Δ12 were expressed in *Escherichia coli* Rosetta2(DE3)pLysS (Novagen). Bacteria were grown in 2× TY medium to an *A*_600_ of 0.8 at 37 °C and cooled to 22 °C, and protein expression was induced by the addition of 0.2 mm isopropyl β-d-thiogalactopyranoside. After 16 h, cells were harvested by centrifugation at 5000 × *g* for 15 min at 4 °C, and the pellet was stored at −20 °C until required.

Cells were thawed and resuspended in 20 mm Tris, 500 mm NaCl, 30 mm imidazole, 1.4 mm β-mercaptoethanol, 0.05% Tween 20, pH 7.5, supplemented with 400 units of bovine DNase I (Sigma-Aldrich) and 200 μl of EDTA-free protease inhibitor mixture (Sigma-Aldrich) before lysis at 165.5 MPa using a TS series cell disruptor (Constant Systems) and centrifugation at 40,000 × *g* for 30 min at 4 °C. Cleared lysate was incubated with Ni^2+^-NTA-agarose (Qiagen) for 1 h at 4 °C, the beads were washed, and the bound protein eluted in 20 mm Tris, 500 mm NaCl, 250 mm imidazole, pH 7.5, before injection onto a Superdex 75 16/600 size exclusion chromatography (SEC) column (GE Healthcare) equilibrated in 20 mm Tris, pH 7.6, 200 mm NaCl, 2 mm DTT (SEC buffer). Purified proteins were concentrated, snap-frozen in liquid nitrogen, and stored at −80 °C until required.

Multiangle light scattering (MALS) experiments were performed at room temperature immediately after SEC at a flow rate of 0.5 ml/min by inline measurement of static light scattering (DAWN 8+, Wyatt Technology), differential refractive index (Optilab T-rEX, Wyatt Technology), and 280 nm absorbance (Agilent 1260 UV, Agilent Technologies). Samples (100 μl of 11.6, 4.1, or 1.2 mg/ml full-length A49; 13.5, 4.7, or 1.4 mg/ml A49 Δ12; and 10.0, 3.5, or 1.0 mg/ml N1) were injected onto an analytical Superdex 75 10/300 gel filtration column (GE Healthcare) equilibrated in SEC buffer. Molar masses were calculated using ASTRA 6 (Wyatt Technology).

##### Crystallization, Structure Solution, Refinement, and Analysis

All crystals were grown by sitting drop vapor diffusion (23) and snap-cryocooled by immersion in liquid nitrogen. Full-length A49 (100 nl at 9.5 mg/ml) was mixed with 100 nl of reservoir solution and equilibrated at 21 °C against 95-μl reservoirs comprising 25% (w/v) PEG 3350, 0.2 m ammonium sulfate, and 0.1 m Tris, pH 9.5. Cryoprotection was achieved by quickly sweeping the crystal through a reservoir supplemented with 20% (v/v) glycerol. A49 Δ12 (1 μl at 25.0 mg/ml) was mixed with 1 μl of reservoir solution and equilibrated at 20 °C against 500-μl reservoirs containing 0.1 m HEPES, pH 7.5, 1.6 m ammonium sulfate, and 1.5% (v/v) PEG 400. Crystals were cryoprotected by passage through 2 μl of perfluoropolyether oil (Hampton Research) that had been overlaid onto the mother liquor. Because the presence of ammonium sulfate in the mother liquor prevented efficient heavy atom derivatization, ammonium sulfate was substituted for sodium malonate (24), and A49 Δ12 crystals were grown by mixing 2 μl of protein (20–21 mg/ml) with 2 μl of reservoir solution and equilibrating at 20 °C against 500-μl reservoirs containing 1.3–1.5 m sodium malonate, pH 6.8, 1% (v/v) PEG 400, and 4–10% (v/v) glycerol, the best crystals being obtained when the reservoir was overlaid with 100 μl of a 1:1 mixture of paraffin and silicone oil (Molecular Dimensions). After crystals had grown, 1 μl of a 1:100 dilution of a saturated potassium dicyanoaurate(I) (KAu(CN)_2_) solution was added to selected drops, and crystals were incubated for 6 days. Crystals were cryoprotected by brief immersion in 2.1 m sodium malonate, 1% (v/v) PEG 400, and 5% (v/v) glycerol.

Diffraction data were collected at 100 K on European Synchrotron Radiation Facility beamline ID14-2 and processed with HKL2000 (full-length A49) or on Diamond Light Source beamline I04-1 and processed with XDS and XSCALE (25), as implemented by xia2 (26) (A49 Δ12). The structure of A49 Δ12 was solved by single-wavelength anomalous dispersion analysis of a potassium dicyanoaurate(I) derivative using the autoSHARP (27) structure solution pipeline. Eight gold sites were identified with occupancy ranging from 0.88 to 0.18. autoSHARP implemented ARP/wARP (28) to build the initial model that was manually improved with COOT (29) and refined using Refmac5 (30). The resultant model was used to solve the structure of A49 Δ12 grown in the sodium malonate condition by molecular replacement with MOLREP (31). This model was manually improved with COOT (29) and refined using Refmac5 (30) with one translation-libration-screw anisotropic displacement group per molecule and local non-crystallographic symmetry restraints. The structures of A49 Δ12 crystallized in the ammonium sulfate condition and of full-length A49 were solved by molecular replacement with MOLREP (31) and PHASER (32), respectively, using the A49 Δ12 sodium malonate structure as a search model and were refined using Refmac5 as detailed above. The stereochemistry of all structures was assessed and improved using COOT, the MolProbity Web server (33), and WHAT_CHECK (34).

A representative set of proteins with the Bcl-2 fold were selected with the assistance of the Pfam Web server (35). Where multiple structures were available for one protein, crystal structures determined at the highest resolution were selected. Where crystal structures had multiple molecules per asymmetric unit, LSQMAN (36) was used to select the molecule that could be superposed on all others with the lowest Cα atom root mean square deviation. For NMR ensembles, the well ordered “core” of the most representative member of the ensemble was selected in consultation with the OLDERADO Web server (37). VACV protein F1, which exists as a domain-swapped dimer both in solution and *in crystallo* (15), was excluded from the analysis. The structural core of the Bcl-2 fold was identified by iterative determination of maximal equivalent substructures, structures were superposed onto this minimal Bcl-2 core, and the resultant superpositions were scored to generate a phylogenetic relationship based on structural equivalence using HSF as described previously (38). Clustering of equivalent substructures and the structure-based phylogenetic tree were visualized using Dendroscope (39). Sequence figures were generate with ALINE (40), molecular graphics were generated using PyMOL (Schroedinger LLC), and images were assembled using Inkscape.

##### Tissue Culture and Viruses

CV-1 and HEK293T cells were cultured in Dulbecco's modified Eagle's medium (DMEM; Invitrogen) supplemented with 10% heat-treated (56 °C, 1 h) FBS (Harlan Sera-Lab), 50 IU/ml penicillin, and 50 μg/ml streptomycin (Invitrogen) at 37 °C in a humidified 5% CO_2_ atmosphere.

To generate vA49-cTAP, the *A49R* ORF plus 213 bp upstream was amplified from VACV WR genomic DNA using oligonucleotides 5′-GTAGGTACCAACAAAAGGTATTACAAGAAT-3′ and 5′-GTAGCGGCCGCCAAATATCGTTCGCGGATATC-3′, containing KpnI and NotI restriction sites (underlined), respectively. The PCR fragment was cloned into a modified pUC13 plasmid upstream of a TAP tag comprising two copies of the streptavidin binding sequence (Strep-tag II) followed by one FLAG epitope (41). The modified pUC13 also contains *E. coli* guanylphosphoribosyl transferase fused in frame with enhanced GFP under the control of a VACV promoter (42). The right-flanking region of *A49R* was amplified using oligonucleotides 5′-GTACCGCGGAAATATTAAAAAAAAATA-3′ and 5′-TCTAGACGGATTTCTGTGTTCTCTTTGAAG-3′, containing SacII and XbaI restriction sites (underlined), respectively, and was cloned downstream of the TAP tag to form pA49vTAP. vA49-cTAP was made by transient dominant selection (43) after infection of CV-1 cells with vΔA49 (5) and transfection with pA49vTAP as described previously (5). VACV WR containing a C-terminally HA-tagged *B14R* (vB14-HA) was described previously (22).

##### Co-immunoprecipitation and Immunoblotting

HEK293T cells were transfected using the calcium phosphate method. Briefly, cells were seeded in 10-cm dishes to reach 50% density after 24 h, at which time the medium was refreshed and cells were allowed to rest for 20 min. DNA (5 μg) was incubated with 50 μl of 2.5 m CaCl_2_ and sterile water to a final volume of 500 μl for 20 min at room temperature. An equal volume of 280 mm NaCl, 10 mm KCl, 1.5 mm Na_2_HPO_4_, 12 mm glucose, 50 mm HEPES, pH 7.05, was added, and the mixture was incubated for a further 15 min. The mixture was dropped carefully onto the cells, and the dishes were swirled gently to distribute it evenly. After 24 h, the medium was removed, and cells were infected (or mock-infected) with the indicated viruses in fresh DMEM with 2% FBS plus penicillin and streptomycin.

To test binding of A49 to proapoptotic Bcl-2 proteins, cells were infected at a multiplicity of infection of 5 for 6 h before harvesting and washing twice with cold PBS. Cells were lysed with CHAPS buffer (20 mm Tris, pH 8, 137 mm NaCl, 2% (w/v) CHAPS, 1 mm EDTA, pH 8, and protease inhibitors (Roche Applied Science)). Lysates were treated with benzonase (Novagen) for 30 min, cleared, and then incubated with anti-FLAG M2 affinity resin (Sigma-Aldrich) for 16 h at 4 °C. After four washes with CHAPS buffer, proteins were boiled off of the beads in 2× SDS-PAGE loading buffer and analyzed by SDS-PAGE and immunoblotting.

To test for A49 self-association, cells were infected at a multiplicity of infection of 2 (multiplicity of infection of 1.5 for each virus when co-infecting) for 16 h before harvesting and washing with cold PBS. Cells were lysed with immunoprecipitation buffer (20 mm Tris, pH 7.4, 150 mm NaCl, 10 mm CaCl_2_, 0.1% (v/v) Triton X-100, 10% (v/v) glycerol, and protease inhibitors (Roche Applied Science)). Cleared lysates were incubated with anti-FLAG M2 affinity resin for 16 h at 4 °C. The resin was washed four times with immunoprecipitation buffer before proteins were boiled off of the beads in 2× SDS-PAGE loading buffer and analyzed by SDS-PAGE and immunoblotting.

Following SDS-PAGE, proteins were transferred onto nitrocellulose membranes and immunoblotted using the Trans-Blot semidry transfer system (Bio-Rad) as per the manufacturer's instructions. Primary antibodies used recognized tubulin (clone DM1A, Upstate Biotechnology), FLAG (F7425, Sigma-Aldrich), HA (mm2-101p (Biolegend) and H6908 (Sigma-Aldrich)), His_6_ (34670, Qiagen), and Myc (9B11, Cell Signaling). The antibody against D8 (AB1.1) was described previously (44). Immunoblots were visualized using an Odyssey scanner (LI-COR Biosciences), except for tubulin (Fig. 2), Myc-β-TrCP (Fig. 3), and HA (Figs. 2 and 3), where enhanced chemiluminescence was used.

## RESULTS

### 

#### 

##### Crystal Structure Reveals That A49 Adopts the Bcl-2 Fold

Crystallization trials of full-length VACV A49 yielded just two crystals in a single crystallization condition after 4 months of equilibration. Diffraction data were recorded to 3.0 Å resolution (full-length A49; Table 1) but, because A49 does not share identifiable sequence similarity with any proteins of known structure, it was not possible to solve the structure by molecular replacement, and repeated attempts to reproduce these crystals were unsuccessful, frustrating attempts to solve the structure by experimental methods. Previous work had shown that the N-terminal 12 amino acids of A49 are essential for its interaction with β-TrCP and share similarity with the β-TrCP-binding sequence of β-catenin (5). The residues of β-catenin that bind β-TrCP have an extended conformation (8), suggesting that the equivalent residues of A49 may be poorly ordered and thus inhibiting crystallization. A truncated form of A49 lacking the N-terminal 12 amino acids (A49 Δ12) was thus expressed and purified. It crystallized readily in two distinct conditions, and diffraction data were recorded to 1.8–1.9 Å resolution (A49 Δ12 ammonium sulfate and A49 Δ12 sodium malonate; Table 1). The structure was solved by single wavelength anomalous dispersion analysis of a A49 Δ12 crystal soaked with potassium dicyanoaurate(I). This initial model was used to solve the structures of full-length A49 and Δ12 A49 in two distinct crystal forms by molecular replacement, which were refined as listed in Table 2.

**TABLE 1:** **Data collection** Values for the highest resolution shell are in parentheses.

	Full-length A49	A49 Δ12 (ammonium sulfate)	A49 Δ12 (sodium malonate)	A49 Δ12 (gold derivative)*^a^*
Beamline	ESRF ID14-2	Diamond I04-1	Diamond I04-1	Diamond I04-1
Wavelength (Å)	0.933	0.920	0.920	0.920
Resolution limits (Å)	50.0-3.0 (3.05-3.00)	45.7-1.8 (1.89-1.84)	33.0-1.9 (1.95-1.90)	27.7-1.7 (1.69-1.65)
Space group	*P*4_3_2_1_2	*P*2_1_	*P*2_1_	*P*2_1_
Cell dimensions				
*a*, *b*, *c* (Å)	67.8, 67.8, 153.9	92.3, 45.6, 160.3	56.9, 42.7, 67.2	79.9, 38.8, 96.9
α, β, γ (degrees)	90.0, 90.0, 90.0	90.0, 98.7, 90.0	90.0, 100.5, 90.0	90.0, 101.4, 90.0
Unique reflections	7753 (365)	112,855 (8107)	25,211 (1842)	70,769 (5232)
Redundancy	13.8 (14.4)	6.8 (6.6)	14.1 (13.5)	24.8 (12.9)
Completeness (%)	100.0 (100.0)	97.9 (96.0)	99.6 (99.0)	99.9 (99.9)
〈*I*/σ(*I*)〉	17.5 (2.2)	13.6 (1.5)	32.2 (2.9)	20.8 (1.9)
CC½	1.000 (0.803)	0.999 (0.719)	1.000 (0.920)	1.000 (0.696)
*R*_merge_	0.101	0.068 (1.160)	0.038 (1.154)	0.127 (1.470)
*R*_pim_	0.045 (0.566)	0.028 (0.483)	0.014 (0.452)	0.024 (0.420)

*^a^* Single-wavelength anomalous dispersion phasing statistics were as follows. SHARP figures of merit for centrics/acentrics were 0.156/0.339 (27.66–1.65 Å), 0.191/0.645 (27.66–7.14 Å), and 0.152/0.169 (1.69–1.65 Å). SHARP anomalous phasing power for acentrics was 0.970 (27.66–1.65 Å), 2.984 (27.66–7.14 Å), and 0.217 (1.69–1.65 Å). Figures of merit after solvent flattening were 0.88 (94.97–1.65 Å), 0.913 (94.97–4.48 Å), and 0.814 (1.68–1.65 Å).

**TABLE 2:** **Refinement Statistics** Values for the highest resolution shell are in parentheses.

	Full-length A49	A49 Δ12 (ammonium sulfate)	A49 Δ12 (sodium malonate)
PDB ID	4D5S	4D5T	4D5R
Resolution limits (Å)	154.0-3.0 (3.08-3.00)	45.7-1.8 (1.89-1.84)	33.0-1.9 (1.95-1.90)
Molecules per asymmetric unit	2	8	2
No. of reflections in working set	7350 (532)	107,126 (7671)	23,928 (1744)
No. of reflections in test set	356 (24)	5665 (414)	1279 (89)
*R*_work_	0.220 (0.358)	0.206 (0.332)	0.185 (0.298)
CC_work_	0.967 (0.732)	0.964 (0.781)	0.974 (0.891)
*R*_free_	0.273 (0.323)	0.239 (0.350)	0.226 (0.286)
CC_free_	0.952 (0.771)	0.952 (0.772)	0.959 (0.902)
CC*^a^	1.000 (0.944)	1.000 (0.915)	1.000 (0.979)
No. of atoms			
Protein	2292	10,096	2442
Water	0	894	85
Other	0	25	0
No. of atoms with alternate conformations	0	764	86
Residues in Ramachandran favored region (%)	91.8	98.6	96.0
Ramachandran outliers (%)	0.7	0.0	0.3
Root mean square deviation			
Bond length (Å)	0.012	0.017	0.018
Bond angle (degrees)	1.370	1.689	1.749
Average B factor (Å^2^)			
Protein	95.0	37.3	62.9
Water		42.6	59.0
Other		43.3	

*^a^* CC*=√(2CC_½_/(1+CC_½_)) (see Ref. 84).

**FIGURE 1.**
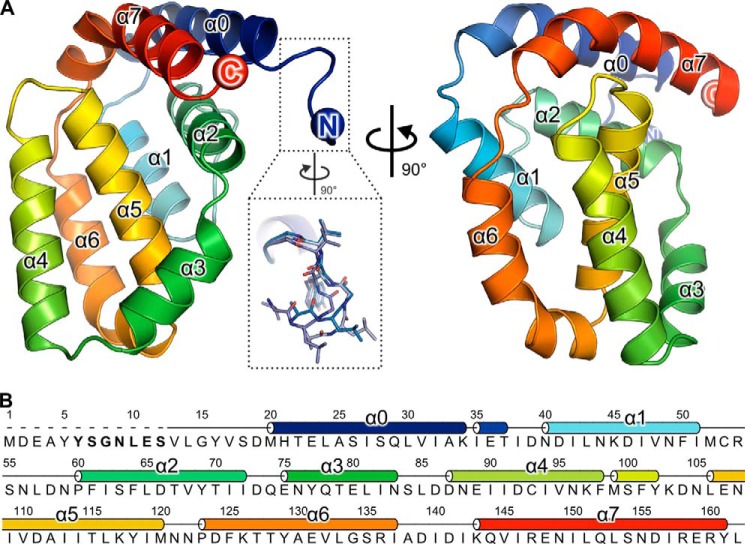
**A49 adopts a Bcl-2-like fold.** *A*, the structure of A49 Δ12 is shown in two *orthogonal views* as a *ribbon colored* from *blue* (N terminus) to *red* (C terminus). The *inset* shows the alternative conformations of residues 13–17 observed, highlighting the mobility of this region. In crystals of A49 grown from full-length protein, no density was observed for these residues. *B*, sequence of VACV WR A49. The secondary structure is shown *above* the sequence, and residues thought to interact with the β-propeller domain of β-TrCP are in *boldface type*.

**FIGURE 2.**
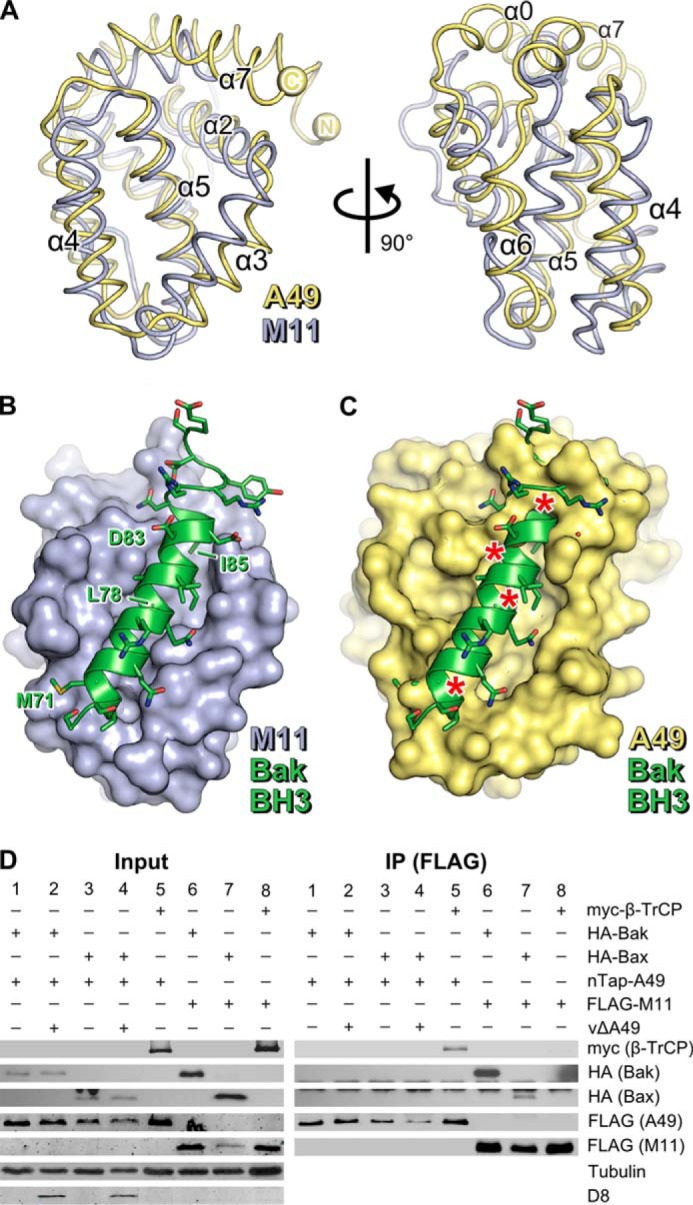
**A49 lacks a surface groove and does not bind BH3 peptides.** *A*, the structures of MYXV M11 (*blue ribbons*, PDB 2JBY (14)) and VACV A49 (*yellow ribbons*) are shown, superposed, in two *orthogonal views*. Selected helices are *labeled. B*, the structure of the human Bak BH3 peptide (*green ribbon* and side chains) bound to M11 (*blue molecular surface*) is shown (PDB 2JBY (14)). *C*, model of A49 in complex with the human Bak BH3 peptide generated by superposing A49 onto the M11-Bak complex (14). *Asterisks* denote significant clashes. *D*, unlike M11, A49 does not co-immunoprecipitate with human Bak or Bax. HEK293T cells were transfected with Myc-β-TrCP (*lanes 5* and *8*), HA-Bak (*lanes 1*, *2*, and *6*), or HA-Bax (*lanes 3*, *4*, and *7*) and nTAP-A49 (*lanes 1–5*) or FLAG-M11 (*lanes 6–8*). After 24 h, cells were infected with vΔA49 at 5 pfu/cell (*lanes 2* and *4*) or mock-infected (*lanes 1*, *3*, and *5–8*). Cells were lysed 6 h after infection, and lysates were immunoprecipitated (*IP*) with an anti-FLAG matrix before immunoblotting using the antibodies specified. Tubulin served as a loading control, and D8, a VACV envelope protein, served as a positive marker of infection.

The structure of A49 Δ12 is presented in Fig. 1, comprising A49 residues 13–162. Strikingly, despite a lack of sequence conservation with known family members, A49 adopts the Bcl-2 family protein fold comprising five α-helices wrapped around a central helix, α5 (45). A search of the PDB using the PDBeFOLD server (46) with A49 as a query identified myxoma virus (MYXV) protein M11 as the closest homologue (3.2 Å root mean square deviation over 92 Cα atoms), despite the fact that the aligned residues share only 8% sequence identity. Whereas helices α1, α2, and α5 of A49 and M11 overlay well, helices α3, α4 and α6 are significantly rotated in A49 compared with their orientations in M11 (Fig. 2*A*). As in VACV protein F1, the Bcl-2 domain of A49 is preceded by an additional helix (α0), but unlike F1, where α0 extends away from the Bcl-2 domain (15), in A49 this helix packs tightly against helices α2 and α7 (Fig. 1). Single turns of the 3_10_ helix immediately follow helices α0 and α4. As with other poxvirus Bcl-2 family proteins, A49 has a single long C-terminal helix (α7) rather than two shorter helices (47), but unlike other poxvirus Bcl-2 proteins, α7 lies parallel to α0 and its C-terminal residues contact residues at the C terminus of α2 (Fig. 1).

The structure of full-length A49 closely resembles that of the truncated protein, superposing on A49 Δ12 with 0.50–0.67 Å root mean square deviation across 140 Cα atoms. In the full-length structure, residues 1–17 could not be modeled due to a lack of interpretable electron density N-terminal to residue 18. It is possible that these amino acids were removed by serendipitous in-drop proteolysis prior to crystallization. Residues 13–17 adopt three distinct conformations in the 10 A49 Δ12 molecules observed in the two crystal forms (Fig. 1*A*, *inset*) and are disordered in one of the two molecules of A49 Δ12 per asymmetric unit in crystals grown in the sodium malonate condition. The only other significant difference between the structures of full-length and Δ12 A49 is the conformation of the region linking helices α2 and α3, helix α3 being one turn shorter in the full-length structure. In four of the eight molecules of A49 Δ12 grown in the ammonium sulfate condition and both molecules of A49 Δ12 grown in the sodium malonate condition, a single residue (Gly) of the C-terminal purification tag is observed; in the structure of full-length A49, the final two residues (161 and 162) and the purification tag were not ordered and could not be modeled.

##### Unlike M11, A49 Does Not Bind Effector BH3 Peptides

A characteristic feature of antiapoptotic Bcl-2-like proteins is their ability to bind amphipathic BH3 peptides via a hydrophobic surface groove delineated by helices α2, α3, α4, and α5 (17). MYXV M11 is a Bcl-2-like protein that inhibits host-cell apoptosis by binding the effector BH3 peptides of proapoptotic Bcl-2 family proteins Bax and Bak (14, 48). Superposition of A49 onto the structure of M11 shows that the positions of helices α2–α5 are generally conserved between the two proteins (Fig. 2*A*). A simple model of A49 bound to a BH3 peptide was generated by superposing A49 Δ12 onto the structure of M11 in complex with the Bak BH3 peptide (14). As shown in Fig. 2, *B* and *C*, although a deep groove is evident on the surface of A49 flanked by helices α2–α5, it is not compatible with the binding of BH3 peptides. The side chain of Bak residue Leu^78^ would clash with the charged side chains of A49 residues Lys^97^ and Asp^111^. Bak residue Leu^78^ interacts with a hydrophobic patch on the M11 surface, is absolutely conserved across BH3 peptides (49) and is essential for strong interaction between BH3 peptides and Bcl-2 family proteins (50). More strikingly, the orientation of A49 helix α7 shortens the groove such that Bak residues 80–90, including a conserved aspartic acid and hydrophobic residue (Asp^83^ and Ile^85^ in Bak, respectively), would clash with A49. Additionally, helix α4 is rotated in A49 relative to its orientation in M11 such that A49 residue Asp^92^ would clash with Bak Met^71^.

Co-immunoprecipitation experiments confirmed the inability of A49 to bind BH3 peptides of the proapoptotic proteins Bax and Bak (Fig. 2*D*). HA-tagged Bax and Bak are co-immunoprecipitated from HEK293T cells by an anti-FLAG affinity matrix when co-expressed with FLAG-tagged M11. These proteins are not co-immunoprecipitated from cells expressing nTAP-A49, where the A49 protein is Strep-tag II- and FLAG-tagged. Concomitant infection by VACV lacking an endogenous *A49R* gene (vΔA49) does not stimulate co-immunoprecipitation of Bax or Bak with A49, confirming that no other viral factor is required for their interaction. Myc-β-TrCP is co-immunoprecipitated with nTAP-A49, confirming that nTAP-A49 is functional (5) and is not co-immunoprecipitated with FLAG-M11, confirming the specificity of the interaction between A49 and β-TrCP.

##### A49 Does Not Dimerize in Solution or in Cells

**FIGURE 3.**
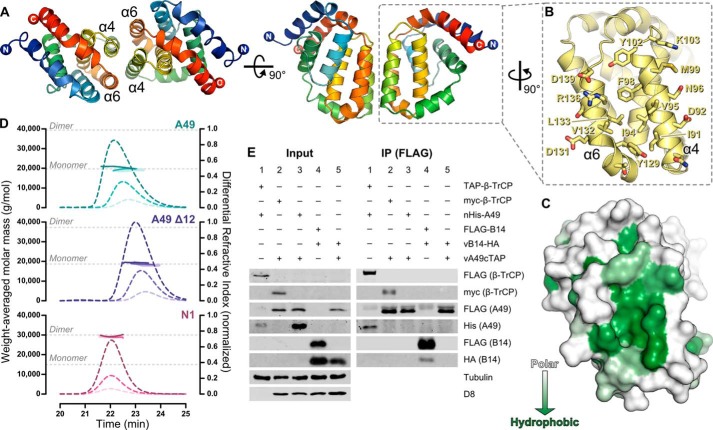
**A49 does not oligomerize in solution or in cells.** *A*, two molecules of A49 Δ12, which interact via helices α4 and α6 (the “4-6 face”) are shown in two *orthogonal views*, *colored* as in Fig. 1*A. B*, the 4-6 face of A49 Δ12, with side chains of residues that form the homotypic contact surface shown as *sticks. C*, molecular *surface representation* of the A49 Δ12 4-6 face *colored* by amino acid hydrophobicity from *white* (polar) to *green* (hydrophobic). *D*, SEC-MALS of full-length A49 (*top*), A49 Δ12 (*middle*), and N1 (*bottom*), each analyzed at three concentrations. Weight-averaged molar masses (*colored solid lines*) are shown across the elution profiles (normalized differential refractive index, *colored dashed lines*). The expected molar masses for monomers and dimers of each protein are shown (*gray dotted lines*). *E*, A49 does not self-associate in cells. HEK293T cells were transfected with TAP-tagged (*lane 1*) or Myc-tagged (*lane 2*) β-TrCP and nHis-A49 (*lanes 1* and *3*) or FLAG-B14 (*lane 4*). After 24 h, cells were mock-infected (*lane 1*) or infected with vA49-cTAP (*lanes 2* and *3*) or vB14-HA (*lane 4*) at 2 pfu/cell or co-infected with both viruses at 1.5 pfu for each virus per cell (*lane 5*). Cells were lysed 16 h after infection, and lysates were immunoprecipitated (*IP*) with an anti-FLAG matrix before immunoblotting using the antibodies specified. Tubulin served as a loading control, and D8 served as a positive marker of infection.

A conserved feature of the VACV Bcl-2-like proteins is their propensity to form dimers; all VACV Bcl-2 family proteins except for K7 crystallize as dimers (11–13, 15, 51, 52). Visual inspection of the structures of A49 Δ12 and full-length A49 showed that, in all cases, two molecules of A49 self-associate via an interaction of helices α4 and α6 (the “4-6 face”; Fig. 3*A*). In the structure of full-length A49 this interaction is formed by crystallographic 2-fold symmetry, whereas in the structures of A49 Δ12 the interaction is formed by pseudo-2-fold non-crystallographic symmetry. The 4-6 face that mediates the interaction comprises a large patch of hydrophobic residues surrounded by a ring of charged and polar amino acids (Fig. 3, *B* and *C*). Whereas the self-association is predominantly hydrophobic in nature, in the structure of full-length A49 a symmetric salt bridge is formed at the periphery of the binding surface between the side chains of Arg^136^ and Asp^92^. In the structures of A49 Δ12 salt bridges Arg^136^–Asp^92^ and Lys^103^–Asp^139^ are formed, although these bonds are not symmetric and thus formed only once per pair of A49 molecules. In A49 Δ12 hydrogen bonds are formed between the side chains of Asn^88^ and Tyr^102^ and the carbonyl oxygens of Thr^128^ and Val^95^, respectively, and between the side chains of Arg^136^ and Asn^96^, although again these interactions often occur only once per pair of A49 molecules. Analysis of the A49 structures using PDBePISA suggested that this 4-6 face plays an essential role in A49 self-association (CSS scores 0.5–1.0) (53). However, previous studies of VACV B14 showed that the self-association interface observed *in crystallo* was only of modest affinity *in vitro* and overlapped with the interface that mediates the interaction between B14 and IκB kinase β (IKKβ) in cells (54). We therefore sought to determine the oligomeric state of A49. SEC-MALS of full-length A49 and A49 Δ12 produced in bacteria showed that both are monomeric across a range of concentrations (8.6–95.1 μm), whereas at even the lowest concentration tested (7.9 μm), VACV N1 is exclusively dimeric (Fig. 3*D*). To test whether A49 can self-associate in cells, HEK293T cells were transfected with nHis-A49 and then infected with vA49-cTAP, where the A49 protein is Strep-tag II- and FLAG-tagged. Following incubation of cell lysate with an anti-FLAG matrix, co-immunoprecipitation of nHis-A49 with A49-cTAP was not observed (Fig. 3*E*, *lane 3*). A49-cTAP and nHis-A49 co-immunoprecipitated with Myc-β-TrCP and TAP-β-TrCP, respectively, confirming that both A49 alleles are functional (Fig. 3*E*, *lanes 1* and *2*). Under the same conditions, HA-tagged B14 co-immunoprecipitates with FLAG-B14 (Fig. 3*E*, *lane 4*) despite the affinity of the B14 self-association being relatively modest (∼20 μm) (54). B14-HA does not co-immunoprecipitate with A49-cTAP, confirming the specificity of the B14 self-association (Fig. 3*E*, *lane 5*). These data indicate that, although crystallized A49 self-associates via the 4-6 face, the protein does not form dimers either in solution or in cells.

##### Relationship of A49 to Viral and Cellular Bcl-2 Family Proteins

Exhaustive searches of the NCBI nr (non-redundant) protein sequence database using PHMMER (55) and HHSENSER (56) yielded homologues of A49 only in orthopoxviruses and Yoka poxvirus. The genes encoding A49 homologues in ectromelia virus, taterapox virus, monkeypox virus, and camelpox virus are all severely truncated and predicted to not give rise to functional proteins. Among other orthopoxviruses A49 is well conserved, all sequences sharing >94% identity with A49 from VACV WR, and the residues of the N peptide predicted to interact with β-TrCP are absolutely conserved (5). The A49 homologue in Yoka poxvirus shares 33% identity with VACV WR A49. Yoka poxvirus A49 has 13 amino acids N-terminal to helix α0, but the sequence of this region differs significantly from that of VACV WR A49.

**FIGURE 4.**
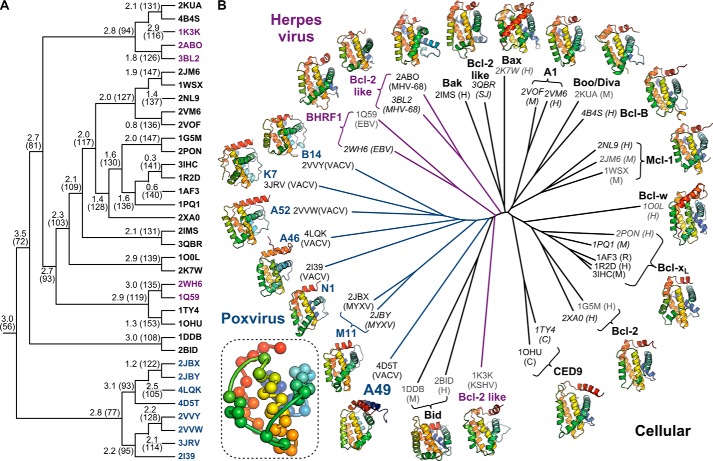
**Poxvirus Bcl-2 proteins are structurally closer to each other than to other cellular or viral Bcl-2 proteins.** *A*, clustering of cellular and viral Bcl-2 proteins to determine the minimal structural “core” of the Bcl-2 fold. PDB identifiers of structures used are shown as *leaves* on the *tree*, *colored* by source organism (*black*, cellular; *magenta*, herpesvirus; *blue*, poxvirus). Cα atom root mean square deviation (Å) and number of equivalent Cα atoms, which corresponds to the size of the common core, are shown at each *branch point*. For *higher branches* in the tree, superpositions were performed between these common cores to iteratively determine the minimal core of the Bcl-2 fold. *B*, structure-based phylogenetic tree showing the relationship between cellular, herpesvirus, and poxvirus Bcl-2 proteins. PDB codes for each structure used are given with their species of origin in *parentheses* (human (*H*), mouse (*M*), rat (*R*), *Caenorhabditis elegans* (*C*), *Schistosoma japonicum* (*SJ*), vaccinia virus (*VACV*), myxoma virus (*MYXV*), Kaposi sarcoma herpesvirus (*KSHV*), murine γ-herpesvirus 68 (*MHV-68*), and Epstein-Barr virus (*EBV*)). Structures determined by x-ray crystallography are *labeled* in *black*, NMR models are shown in *gray*, and structures determined with BH3 peptides or C-terminal tails bound in the surface groove are *italicized. Ribbon diagrams* of representative structures for each protein are shown, *color-ramped* from *blue* (N terminus) to *red* (C terminus). The *inset* shows the 56 Cα atoms that comprise the minimal core of the Bcl-2 fold, *colored* as above. Coordinate files containing the core Cα atoms of the Bcl-2 fold plus superpositions of the 35 representative Bcl-2 family structures upon this core are supplied as supplemental material.

The three-dimensional structures of distantly related proteins are more highly conserved than their amino acid sequences (57). Given the high sequence divergence between A49 and other poxvirus Bcl-2-like proteins, we wondered whether A49 was structurally closer to poxvirus, herpesvirus, or cellular Bcl-2 family proteins. A representative set of 23 cellular, 5 herpesvirus, and 7 poxvirus Bcl-2 family protein structures, solved in the presence or absence of protein binding partners, was assembled and then aligned and clustered by iterative pairwise comparison of their structural and physicochemical properties as described previously (38). Briefly, pairwise comparison of structures was used to identify the set of equivalent residues that defines their common structural “core,” and the two most closely related structures (or structural cores) were merged. This process was repeated until only one core was left, producing the hierarchical clustering of structural similarity shown in Fig. 4*A* and identifying the minimal set of amino acid residues that defines the Bcl-2 fold. This minimal core, comprising 56 residues, spans the majority of helices α1, α2, and α5 but also comprises elements of α3, α6 and α7 (Fig. 4*B*, *inset*). All structures were aligned upon this minimal core and all-pairs pairwise comparison of the structures was performed as described previously (38) to generate the phylogenetic tree shown in Fig. 4*B*. Strikingly, despite the lack of sequence conservation, the structures of poxvirus Bcl-2 family proteins, including A49, more closely resemble each other than they do cellular or herpesvirus Bcl-2-like proteins, consistent with previous analysis of the poxvirus proteins A52, B14, M11, and N1 (13). A49 lies closest on the tree to MYXV M11 and VACV N1. Both N1 and M11 bind BH3 peptides and inhibit apoptosis (12, 18, 48), although N1 does so only weakly compared with other VACV antiapoptotic proteins (58). The other VACV proteins that inhibit cellular innate immune responses (A52, K7, B14, and A46) all cluster together despite having a diverse range of cellular binding partners (12, 13, 59–63). This is in contrast to cellular Bcl-2 proteins, where orthologues that share a function lie closer to each other on the tree than paralogues from the same species with different functions. Overall, the cellular proteins partition away from the virus proteins with the exception of Bid, which falls between the herpesvirus and poxvirus Bcl-2 proteins. Bid is unlike other cellular Bcl-2 family proteins in that it has only one Bcl-2 homology domain, BH3, and cleavage of Bid changes its conformation to promote apoptosis (64–66). Because its structure is highly divergent from both cellular and viral Bcl-2 proteins (Fig. 4*A*), its position in the tree probably arises from “long branch attraction” (67) rather than greater structural similarity to viral Bcl-2 proteins.

## DISCUSSION

We expressed recombinant VACV A49 and solved its structure to 1.8 Å resolution (Fig. 1). Surprisingly, A49 adopts a Bcl-2-like fold, despite lacking identifiable sequence similarity with other members of the Bcl-2 family. The defining characteristic of cellular Bcl-2 family proteins is their involvement in the regulation of apoptosis. Intrinsic apoptosis is triggered when the proapoptotic Bcl-2 family effector proteins Bak and Bax oligomerize and permeabilize the outer mitochondrial membrane, leading to an irreversible caspase cascade and cell death (17). Antiapoptotic Bcl-2 proteins oppose apoptosis via a surface groove formed by helices α2–α5. This groove binds to the exposed BH3 peptides of activated Bax or Bak, preventing their oligomerization. The groove also binds the BH3 peptides of BH3-only proteins like Bim, sequestering such peptides and thereby preventing them from binding to and activating Bax or Bak (17). MXYV protein M11 and VACV proteins N1 and F1 all adopt the Bcl-2 fold and inhibit apoptosis by binding BH3 peptides via a groove on their surface formed by helices α2–α5 (12, 14, 15, 18). The A49 structure shows that A49 lacks a surface groove compatible with binding BH3 peptides and is unable to bind the proapoptotic proteins Bax or Bak (Fig. 2). This indicates that unlike M11, its closest structural relative, A49 does not function to inhibit apoptosis by sequestering effector BH3 peptides.

A49 inhibits NF-κB activation by inhibiting ubiquitination and subsequent degradation of IκBα (5). A49 achieves this by sequestering the E3 ligase β-TrCP, preventing it from binding the phosphorylated form of IκBα. The interaction with β-TrCP requires A49 residues 6–12 (5), which contain a double serine motif that is likely to be phosphorylated and bind the β-propeller domain of β-TrCP in an extended conformation similar to that observed in the complex of β-TrCP with β-catenin (5, 8). In the structure of full-length A49, we did not observe any interpretable electron density N-terminal to residue 18, nor did we observe “additional” electron density anywhere in the structure that could be interpreted as residues 1–17. Additionally, we observed that A49 crystallized much more readily upon removal of residues 1–12. These observations indicate that the N-terminal β-TrCP-binding residues of A49 lack intrinsic structure and are thus freely available to bind β-TrCP. However, we note that residues Ser^7^ and Ser^12^ of full-length A49 produced in *E. coli* are unlikely to have been phosphorylated, which may possibly influence the conformation of this region.

The HIV-1 immunomodulatory protein Vpu functions similarly to A49, binding β-TrCP and preventing degradation of IκBα (68). However, Vpu also binds the HIV cell surface receptor CD4 and the restriction factor tetherin, promoting their ubiquitination and degradation by bringing them in close proximity to the SCF^β-TrCP^ E3 ligase complex (69, 70). Although a cellular target of A49-mediated proteasomal or lysosomal degradation has yet to be identified, inspection of the A49 crystal structures identified a surface of the protein that is a prime candidate for mediating such interactions. In all crystal forms presented here the two molecules of A49 self-associate via a symmetric interaction between helices α4 and α6 (the 4-6 face). This interaction is similar to the self-association observed in crystals of VACV A46 (51) but, unlike A46, A49 does not form dimers in solution or in cells (Fig. 3). Inspection of the 4-6 face reveals a hydrophobic surface cleft that would seem ideal for mediating interactions with cellular binding partners (Fig. 3). Dimerization of VACV proteins *in crystallo* by surfaces that mediate binding to cellular partners has been observed before; the 1-6 face of B14 that mediates its reversible self-association in solution overlaps with its binding site for IKKβ (54), mutations that abolish dimerization of N1 also disrupt its ability to inhibit NF-κB activation (18), and a residue of A52 required for binding to TRAF6 lies partly buried within the dimerization interface (71), although in this case maintaining A52 dimerization seems to be required for efficient TRAF6-mediated stimulation of p38 and subsequent induction of IL-10 expression. It is tempting to speculate that A49 binds other cellular factors via the 4-6 face to promote their β-TrCP-mediated ubiquitination and degradation, although further experiments are required to probe this hypothesis.

The structure of A49 takes to 11 the number of VACV proteins that have been shown or predicted to share the Bcl-2 fold (Table 3). Although the bulk of poxvirus Bcl-2 proteins share weak but identifiable sequence similarity (16, 72), A49 could not be identified as a Bcl-2 family protein based on sequence alone. Nonetheless, structure-based phylogenetic analysis shows A49 to be more closely related to poxvirus than herpesvirus or cellular Bcl-2 proteins (Fig. 4). This is consistent with poxvirus Bcl-2 family proteins having arisen from gene duplication and divergence following a single gene acquisition event, structural similarity having been conserved despite vast sequence divergence. The terminal regions of the poxvirus genome are highly variable, containing non-essential genes that act to determine host range and inhibit the host immune response (2). In addition to terminal transpositions, whereby genes from one end of the linear genome are duplicated at the other end (72–74), a recent study showed that poxviruses deploy “genomic accordions” when under selective pressure, their genomes rapidly expanding to incorporate multiple copies of genes near points of genomic instability (75). This expansion increases the probability that duplicated genes will acquire “advantageous” mutations, potentially conferring a divergent function upon the mutated gene. The host innate immune response places large DNA viruses under significant selective pressure (76), and poxvirus Bcl-2 family proteins all act to inhibit the cellular responses to infection. Inspection of the low frequency duplications observed in the Copenhagen strain of VACV (or strains derived therefrom) (75) show independent gene duplications encompassing several Bcl-2 family proteins: the duplicated VACV Copenhagen region spanning nucleotides 143,153–156,405 contains A46 and A49; the duplicated region spanning nucleotides 22,298–29,837 contains C1, N1, and N2; and the duplicated regions spanning nucleotides 24,975–47,387 or 25,066–47,467 contain N1, K7, and F1. This is consistent with the gene duplication and differentiation events that gave rise to the 11 VACV Bcl-2 family immunomodulatory proteins having arisen as a result of ancestral poxviruses deploying their genomic accordions in response to selective pressure generated by adaptation in the host or changes in host range. Although A49 has high sequence divergence from the other poxvirus Bcl-2 proteins, its absence from *Yata*-, *Lepri*-, *Sui*-, *Cervid*-, or *Capripoxviruses*, all of which have multiple Bcl-2 like proteins, makes it a poor candidate for being the *BCL2* gene acquired originally by the ancestral poxvirus.

**TABLE 3:** **VACV proteins with a Bcl-2-like fold**

Protein*^a^*	Function	References
C16/B22	Unknown	13
C6	Inhibition of IRF3 and IRF7	13, 77
C1	Unknown	16
N1	Inhibition of NF-κB and apoptosis	11, 12, 63
N2	Inhibition of IRF3	16, 78
K7	Inhibition of NF-κB and IRF3	13, 62, 79
F1	Inhibition of apoptosis and IL-1β production	15, 80–82
A46	Inhibition of MAPKs, NF-κB and IRF3	13, 51, 61, 83
A49	Inhibition of NF-κB	5, this study
A52	Inhibition of NF-κB and stimulation of p38 MAPK	13, 60, 71, 83
B14*^b^*	Inhibition of NF-κB	13, 59

*^a^* Protein names are for VACV strain Copenhagen except as noted.

*^b^* Encoded by VACV WR gene *B14R*, which is equivalent to VACV Copenhagen gene *B15R* (22).

In summary, we have shown that A49 is an unanticipated eleventh member of the VACV Bcl-2-like immunomodulatory protein family. A49 lacks a BH3 peptide binding groove and does not bind the proapoptotic proteins Bax and Bak. Although A49 self-associates via a hydrophobic 4-6 face in crystals, it does not self-associate in solution or in cells, suggesting that this 4-6 face may mediate binding to yet undetermined cellular partners. Conservation of the Bcl-2 fold by poxvirus proteins with highly divergent sequences is consistent with duplication and divergence of an ancestral gene encoding a Bcl-2 family protein.

## Supplementary Material

Supplemental Data

## Footnotes

3 The abbreviations used are:

VACV: vaccinia virus
IKKβ: IκB kinase β
MALS: multiangle light scattering
MYXV: myxoma virus
SEC: size exclusion chromatography
WR: Western Reserve.

## References

[1] Moss B. (2007) in Fields' Virology, 5th Ed (Fields B. N. Knipe D. M. Howley P. M., eds) pp. 2905–2946, Wolters Kluwer Health/Lippincott Williams & Wilkins, Philadelphia

[2] Gubser C. Hué S. Kellam P. Smith G. L. (2004) Poxvirus genomes: a phylogenetic analysis. J. Gen. Virol. 85, 105–117 14718625 10.1099/vir.0.19565-0

[3] Ferguson B. J. Mansur D. S. Peters N. E. Ren H. Smith G. L. (2012) DNA-PK is a DNA sensor for IRF-3-dependent innate immunity. eLife 1, e00047 23251783 10.7554/eLife.00047 PMC3524801

[4] Walsh S. R. Dolin R. (2011) Vaccinia viruses: vaccines against smallpox and vectors against infectious diseases and tumors. Expert Rev. Vaccines 10, 1221–1240 21854314 10.1586/erv.11.79 PMC3223417

[5] Mansur D. S. Maluquer de Motes C. Unterholzner L. Sumner R. P. Ferguson B. J. Ren H. Strnadova P. Bowie A. G. Smith G. L. (2013) Poxvirus targeting of E3 ligase β-TrCP by molecular mimicry: a mechanism to inhibit NF-κB activation and promote immune evasion and virulence. PLoS Pathog. 9, e1003183 23468625 10.1371/journal.ppat.1003183 PMC3585151

[6] Oeckinghaus A. Ghosh S. (2009) The NF-κB family of transcription factors and its regulation. Cold Spring Harb. Perspect. Biol. 1, a000034 20066092 10.1101/cshperspect.a000034 PMC2773619

[7] Balachandran S. Beg A. A. (2011) Defining emerging roles for NF-kappaB in antivirus responses: revisiting the interferon-β enhanceosome paradigm. PLoS Pathog. 7, e1002165 22022260 10.1371/journal.ppat.1002165 PMC3192840

[8] Wu G. Xu G. Schulman B. A. Jeffrey P. D. Harper J. W. Pavletich N. P. (2003) Structure of a β-TrCP1-Skp1-β-catenin complex: destruction motif binding and lysine specificity of the SCF^β-TrCP1^ ubiquitin ligase. Mol. Cell 11, 1445–1456 12820959 10.1016/s1097-2765(03)00234-x

[9] Smith G. L. Benfield C. T. Maluquer de Motes C. Mazzon M. Ember S. W. Ferguson B. J. Sumner R. P. (2013) Vaccinia virus immune evasion: mechanisms, virulence and immunogenicity. J. Gen. Virol. 94, 2367–2392 23999164 10.1099/vir.0.055921-0

[10] van Buuren N. Burles K. Schriewer J. Mehta N. Parker S. Buller R. M. Barry M. (2014) EVM005: an ectromelia-encoded protein with dual roles in NF-κB inhibition and virulence. PLoS Pathog. 10, e1004326 25122471 10.1371/journal.ppat.1004326 PMC4133408

[11] Aoyagi M. Zhai D. Jin C. Aleshin A. E. Stec B. Reed J. C. Liddington R. C. (2007) Vaccinia virus N1L protein resembles a B cell lymphoma-2 (Bcl-2) family protein. Protein Sci. 16, 118–124 17123957 10.1110/ps.062454707 PMC2222835

[12] Cooray S. Bahar M. W. Abrescia N. G. McVey C. E. Bartlett N. W. Chen R. A. Stuart D. I. Grimes J. M. Smith G. L. (2007) Functional and structural studies of the vaccinia virus virulence factor N1 reveal a Bcl-2-like anti-apoptotic protein. J. Gen. Virol. 88, 1656–1666 17485524 10.1099/vir.0.82772-0 PMC2885619

[13] Graham S. C. Bahar M. W. Cooray S. Chen R. A. Whalen D. M. Abrescia N. G. Alderton D. Owens R. J. Stuart D. I. Smith G. L. Grimes J. M. (2008) Vaccinia virus proteins A52 and B14 Share a Bcl-2-like fold but have evolved to inhibit NF-κB rather than apoptosis. PLoS Pathog. 4, e1000128 18704168 10.1371/journal.ppat.1000128 PMC2494871

[14] Kvansakul M. van Delft M. F. Lee E. F. Gulbis J. M. Fairlie W. D. Huang D. C. Colman P. M. (2007) A structural viral mimic of prosurvival Bcl-2: a pivotal role for sequestering proapoptotic Bax and Bak. Mol. Cell 25, 933–942 17386268 10.1016/j.molcel.2007.02.004

[15] Kvansakul M. Yang H. Fairlie W. D. Czabotar P. E. Fischer S. F. Perugini M. A. Huang D. C. Colman P. M. (2008) Vaccinia virus anti-apoptotic F1L is a novel Bcl-2-like domain-swapped dimer that binds a highly selective subset of BH3-containing death ligands. Cell Death Differ. 15, 1564–1571 18551131 10.1038/cdd.2008.83

[16] González J. M. Esteban M. (2010) A poxvirus Bcl-2-like gene family involved in regulation of host immune response: sequence similarity and evolutionary history. Virol. J. 7, 59 20230632 10.1186/1743-422X-7-59 PMC2907574

[17] Czabotar P. E. Lessene G. Strasser A. Adams J. M. (2014) Control of apoptosis by the BCL-2 protein family: implications for physiology and therapy. Nat. Rev. Mol. Cell Biol. 15, 49–63 24355989 10.1038/nrm3722

[18] Maluquer de Motes C. Cooray S. Ren H. Almeida G. M. McGourty K. Bahar M. W. Stuart D. I. Grimes J. M. Graham S. C. Smith G. L. (2011) Inhibition of apoptosis and NF-κB activation by vaccinia protein N1 occur via distinct binding surfaces and make different contributions to virulence. PLoS Pathog. 7, e1002430 22194685 10.1371/journal.ppat.1002430 PMC3240604

[19] Berrow N. S. Alderton D. Sainsbury S. Nettleship J. Assenberg R. Rahman N. Stuart D. I. Owens R. J. (2007) A versatile ligation-independent cloning method suitable for high-throughput expression screening applications. Nucleic Acids Res. 35, e45 17317681 10.1093/nar/gkm047 PMC1874605

[20] Teo H. Perisic O. González B. Williams R. L. (2004) ESCRT-II, an endosome-associated complex required for protein sorting: crystal structure and interactions with ESCRT-III and membranes. Dev. Cell 7, 559–569 15469844 10.1016/j.devcel.2004.09.003

[21] Bartlett N. Symons J. A. Tscharke D. C. Smith G. L. (2002) The vaccinia virus N1L protein is an intracellular homodimer that promotes virulence. J. Gen. Virol. 83, 1965–1976 12124460 10.1099/0022-1317-83-8-1965

[22] Chen R. A. Jacobs N. Smith G. L. (2006) Vaccinia virus strain Western Reserve protein B14 is an intracellular virulence factor. J. Gen. Virol. 87, 1451–1458 16690909 10.1099/vir.0.81736-0

[23] Walter T. S. Diprose J. M. Mayo C. J. Siebold C. Pickford M. G. Carter L. Sutton G. C. Berrow N. S. Brown J. Berry I. M. Stewart-Jones G. B. Grimes J. M. Stammers D. K. Esnouf R. M. Jones E. Y. Owens R. J. Stuart D. I. Harlos K. (2005) A procedure for setting up high-throughput nanolitre crystallization experiments. Crystallization workflow for initial screening, automated storage, imaging and optimization. Acta Crystallogr. D Biol. Crystallogr. 61, 651–657 15930615 10.1107/S0907444905007808 PMC7159505

[24] Holyoak T. Fenn T. D. Wilson M. A. Moulin A. G. Ringe D. Petsko G. A. (2003) Malonate: a versatile cryoprotectant and stabilizing solution for salt-grown macromolecular crystals. Acta Crystallogr. D Biol. Crystallogr. 59, 2356–2358 14646118 10.1107/s0907444903021784

[25] Kabsch W. (2010) XDS. Acta Crystallogr. D Biol. Crystallogr. 66, 125–132 20124692 10.1107/S0907444909047337 PMC2815665

[26] Winter G. (2010) xia2: an expert system for macromolecular crystallography data reduction. J. Appl. Cryst. 43, 186–190

[27] Vonrhein C. Blanc E. Roversi P. Bricogne G. (2007) Automated structure solution with autoSHARP. Methods Mol. Biol. 364, 215–230 17172768 10.1385/1-59745-266-1:215

[28] Perrakis A. Morris R. Lamzin V. S. (1999) Automated protein model building combined with iterative structure refinement. Nat. Struct. Biol. 6, 458–463 10331874 10.1038/8263

[29] Emsley P. Lohkamp B. Scott W. G. Cowtan K. (2010) Features and development of Coot. Acta Crystallogr. D Biol. Crystallogr. 66, 486–501 20383002 10.1107/S0907444910007493 PMC2852313

[30] Murshudov G. N. Skubák P. Lebedev A. A. Pannu N. S. Steiner R. A. Nicholls R. A. Winn M. D. Long F. Vagin A. A. (2011) REFMAC5 for the refinement of macromolecular crystal structures. Acta Crystallogr. D Biol. Crystallogr. 67, 355–367 21460454 10.1107/S0907444911001314 PMC3069751

[31] Vagin A. Teplyakov A. (1997) MOLREP: an automated program for molecular replacement. J. Appl. Cryst. 30, 1022–1025

[32] McCoy A. J. Grosse-Kunstleve R. W. Adams P. D. Winn M. D. Storoni L. C. Read R. J. (2007) Phaser crystallographic software. J. Appl. Cryst. 40, 658–674 19461840 10.1107/S0021889807021206 PMC2483472

[33] Davis I. W. Leaver-Fay A. Chen V. B. Block J. N. Kapral G. J. Wang X. Murray L. W. Arendall W. B. 3rd Snoeyink J. Richardson J. S. Richardson D. C. (2007) MolProbity: all-atom contacts and structure validation for proteins and nucleic acids. Nucleic Acids Res. 35, W375–W383 17452350 10.1093/nar/gkm216 PMC1933162

[34] Hooft R. W. W. Vriend G. Sander C. Abola E. E. (1996) Errors in protein structures. Nature 381, 272–272 8692262 10.1038/381272a0

[35] Finn R. D. Bateman A. Clements J. Coggill P. Eberhardt R. Y. Eddy S. R. Heger A. Hetherington K. Holm L. Mistry J. Sonnhammer E. L. Tate J. Punta M. (2014) Pfam: the protein families database. Nucleic Acids Res. 42, D222–D230 24288371 10.1093/nar/gkt1223 PMC3965110

[36] Kleywegt G. J. Jones T. A. (1997) Detecting folding motifs and similarities in protein structures. Methods Enzymol. 277, 525–545 18488323 10.1016/s0076-6879(97)77029-0

[37] Kelley L. A. Sutcliffe M. J. (1997) OLDERADO: on-line database of ensemble representatives and domains. Protein Sci. 6, 2628–2630 9416612 10.1002/pro.5560061215 PMC2143626

[38] Ravantti J. Bamford D. Stuart D. I. (2013) Automatic comparison and classification of protein structures. J. Struct. Biol. 183, 47–56 23707633 10.1016/j.jsb.2013.05.007

[39] Huson D. H. Scornavacca C. (2012) Dendroscope 3: an interactive tool for rooted phylogenetic trees and networks. Syst. Biol. 61, 1061–1067 22780991 10.1093/sysbio/sys062

[40] Bond C. S. Schüttelkopf A. W. (2009) ALINE: a WYSIWYG protein-sequence alignment editor for publication-quality alignments. Acta Crystallogr. D Biol. Crystallogr. 65, 510–512 19390156 10.1107/S0907444909007835

[41] Gloeckner C. J. Boldt K. Schumacher A. Roepman R. Ueffing M. (2007) A novel tandem affinity purification strategy for the efficient isolation and characterisation of native protein complexes. Proteomics 7, 4228–4234 17979178 10.1002/pmic.200700038

[42] Ember S. W. Ren H. Ferguson B. J. Smith G. L. (2012) Vaccinia virus protein C4 inhibits NF-κB activation and promotes virus virulence. J. Gen. Virol. 93, 2098–2108 22791606 10.1099/vir.0.045070-0 PMC3541790

[43] Falkner F. G. Moss B. (1990) Transient dominant selection of recombinant vaccinia viruses. J. Virol. 64, 3108–3111 2159565 10.1128/jvi.64.6.3108-3111.1990 PMC249504

[44] Parkinson J. E. Smith G. L. (1994) Vaccinia virus gene A36R encodes a *M*_r_ 43–50 K protein on the surface of extracellular enveloped virus. Virology 204, 376–390 8091668 10.1006/viro.1994.1542

[45] Lama D. Sankararamakrishnan R. (2010) Identification of core structural residues in the sequentially diverse and structurally homologous Bcl-2 family of proteins. Biochemistry 49, 2574–2584 20141168 10.1021/bi100029k

[46] Krissinel E. Henrick K. (2004) Secondary-structure matching (SSM), a new tool for fast protein structure alignment in three dimensions. Acta Crystallogr. D Biol. Crystallogr. 60, 2256–2268 15572779 10.1107/S0907444904026460

[47] Kvansakul M. Hinds M. G. (2013) Structural biology of the Bcl-2 family and its mimicry by viral proteins. Cell Death Dis. 4, e909 24201808 10.1038/cddis.2013.436 PMC3847314

[48] Wang G. Barrett J. W. Nazarian S. H. Everett H. Gao X. Bleackley C. Colwill K. Moran M. F. McFadden G. (2004) Myxoma virus M11L prevents apoptosis through constitutive interaction with Bak. J. Virol. 78, 7097–7111 15194786 10.1128/JVI.78.13.7097-7111.2004 PMC421673

[49] Day C. L. Smits C. Fan F. C. Lee E. F. Fairlie W. D. Hinds M. G. (2008) Structure of the BH3 domains from the p53-inducible BH3-only proteins Noxa and Puma in complex with Mcl-1. J. Mol. Biol. 380, 958–971 18589438 10.1016/j.jmb.2008.05.071

[50] Sattler M. Liang H. Nettesheim D. Meadows R. P. Harlan J. E. Eberstadt M. Yoon H. S. Shuker S. B. Chang B. S. Minn A. J. Thompson C. B. Fesik S. W. (1997) Structure of Bcl-xL-Bak peptide complex: recognition between regulators of apoptosis. Science 275, 983–986 9020082 10.1126/science.275.5302.983

[51] Fedosyuk S. Grishkovskaya I. de Almeida Ribeiro E. Jr. Skern T. (2014) Characterization and structure of the vaccinia virus NF-κB antagonist A46. J. Biol. Chem. 289, 3749–3762 24356965 10.1074/jbc.M113.512756 PMC3916572

[52] Oda S. Schröder M. Khan A. R. (2009) Structural basis for targeting of human RNA helicase DDX3 by poxvirus protein K7. Structure 17, 1528–1537 19913487 10.1016/j.str.2009.09.005

[53] Krissinel E. Henrick K. (2007) Inference of macromolecular assemblies from crystalline state. J. Mol. Biol. 372, 774–797 17681537 10.1016/j.jmb.2007.05.022

[54] Benfield C. T. Mansur D. S. McCoy L. E. Ferguson B. J. Bahar M. W. Oldring A. P. Grimes J. M. Stuart D. I. Graham S. C. Smith G. L. (2011) Mapping the IκB kinase β (IKKβ)-binding interface of the B14 protein, a vaccinia virus inhibitor of IKKβ-mediated activation of nuclear factor κB. J. Biol. Chem. 286, 20727–20735 21474453 10.1074/jbc.M111.231381 PMC3121528

[55] Finn R. D. Clements J. Eddy S. R. (2011) HMMER web server: interactive sequence similarity searching. Nucleic Acids Res. 39, W29–W37 21593126 10.1093/nar/gkr367 PMC3125773

[56] Söding J. Remmert M. Biegert A. Lupas A. N. (2006) HHsenser: exhaustive transitive profile search using HMM-HMM comparison. Nucleic Acids Res. 34, W374–W378 16845029 10.1093/nar/gkl195 PMC1538784

[57] Rossmann M. G. Argos P. (1976) Exploring structural homology of proteins. J. Mol. Biol. 105, 75–95 186608 10.1016/0022-2836(76)90195-9

[58] Veyer D. L. Maluquer de Motes C. Sumner R. P. Ludwig L. Johnson B. F. Smith G. L. (2014) Analysis of the anti-apoptotic activity of four vaccinia virus proteins demonstrates that B13 is the most potent inhibitor in isolation and during viral infection. J. Gen. Virol. 95, 2757–2768 25090990 10.1099/vir.0.068833-0 PMC4233632

[59] Chen R. A. Ryzhakov G. Cooray S. Randow F. Smith G. L. (2008) Inhibition of IκB kinase by vaccinia virus virulence factor B14. PLoS Pathog. 4, e22 18266467 10.1371/journal.ppat.0040022 PMC2233672

[60] Harte M. T. Haga I. R. Maloney G. Gray P. Reading P. C. Bartlett N. W. Smith G. L. Bowie A. O'Neill L. A. (2003) The poxvirus protein A52R targets Toll-like receptor signaling complexes to suppress host defense. J. Exp. Med. 197, 343–351 12566418 10.1084/jem.20021652 PMC2193841

[61] Stack J. Haga I. R. Schröder M. Bartlett N. W. Maloney G. Reading P. C. Fitzgerald K. A. Smith G. L. Bowie A. G. (2005) Vaccinia virus protein A46R targets multiple Toll-like-interleukin-1 receptor adaptors and contributes to virulence. J. Exp. Med. 201, 1007–1018 15767367 10.1084/jem.20041442 PMC2213104

[62] Schröder M. Baran M. Bowie A. G. (2008) Viral targeting of DEAD box protein 3 reveals its role in TBK1/IKKϵ-mediated IRF activation. EMBO J. 27, 2147–2157 18636090 10.1038/emboj.2008.143 PMC2516890

[63] DiPerna G. Stack J. Bowie A. G. Boyd A. Kotwal G. Zhang Z. Arvikar S. Latz E. Fitzgerald K. A. Marshall W. L. (2004) Poxvirus protein N1L targets the I-κB kinase complex, inhibits signaling to NF-κB by the tumor necrosis factor superfamily of receptors, and inhibits NF-κB and IRF3 signaling by Toll-like receptors. J. Biol. Chem. 279, 36570–36578 15215253 10.1074/jbc.M400567200

[64] Li H. Zhu H. Xu C. J. Yuan J. (1998) Cleavage of BID by caspase 8 mediates the mitochondrial damage in the Fas pathway of apoptosis. Cell 94, 491–501 9727492 10.1016/s0092-8674(00)81590-1

[65] Luo X. Budihardjo I. Zou H. Slaughter C. Wang X. (1998) Bid, a Bcl2 interacting protein, mediates cytochrome *c* release from mitochondria in response to activation of cell surface death receptors. Cell 94, 481–490 9727491 10.1016/s0092-8674(00)81589-5

[66] Wang Y. Tjandra N. (2013) Structural insights of tBid, the caspase-8-activated Bid, and its BH3 domain. J. Biol. Chem. 288, 35840–35851 24158446 10.1074/jbc.M113.503680 PMC3861634

[67] Bergsten J. (2005) A review of long-branch attraction. Cladistics 21, 163–193 10.1111/j.1096-0031.2005.00059.x 34892859

[68] Bour S. Perrin C. Akari H. Strebel K. (2001) The human immunodeficiency virus type 1 Vpu protein inhibits NF-κB activation by interfering with β TrCP-mediated degradation of IκB. J. Biol. Chem. 276, 15920–15928 11278695 10.1074/jbc.M010533200

[69] Douglas J. L. Viswanathan K. McCarroll M. N. Gustin J. K. Früh K. Moses A. V. (2009) Vpu directs the degradation of the human immunodeficiency virus restriction factor BST-2/Tetherin via a βTrCP-dependent mechanism. J. Virol. 83, 7931–7947 19515779 10.1128/JVI.00242-09 PMC2715753

[70] Margottin F. Bour S. P. Durand H. Selig L. Benichou S. Richard V. Thomas D. Strebel K. Benarous R. (1998) A novel human WD protein, h-β TrCp, that interacts with HIV-1 Vpu connects CD4 to the ER degradation pathway through an F-box motif. Mol. Cell 1, 565–574 9660940 10.1016/s1097-2765(00)80056-8

[71] Stack J. Hurst T. P. Flannery S. M. Brennan K. Rupp S. Oda S. Khan A. R. Bowie A. G. (2013) Poxviral protein A52 stimulates p38 mitogen-activated protein kinase (MAPK) activation by causing tumor necrosis factor receptor-associated factor 6 (TRAF6) self-association leading to transforming growth factor β-activated kinase 1 (TAK1) recruitment. J. Biol. Chem. 288, 33642–33653 24114841 10.1074/jbc.M113.485490 PMC3837111

[72] Smith G. L. Chan Y. S. Howard S. T. (1991) Nucleotide sequence of 42 kbp of vaccinia virus strain WR from near the right inverted terminal repeat. J. Gen. Virol. 72, 1349–1376 2045793 10.1099/0022-1317-72-6-1349

[73] Kotwal G. J. Moss B. (1988) Analysis of a large cluster of nonessential genes deleted from a vaccinia virus terminal transposition mutant. Virology 167, 524–537 2849238

[74] Pickup D. J. Ink B. S. Parsons B. L. Hu W. Joklik W. K. (1984) Spontaneous deletions and duplications of sequences in the genome of cowpox virus. Proc. Natl. Acad. Sci. U.S.A. 81, 6817–6821 6093123 10.1073/pnas.81.21.6817 PMC392023

[75] Elde N. C. Child S. J. Eickbush M. T. Kitzman J. O. Rogers K. S. Shendure J. Geballe A. P. Malik H. S. (2012) Poxviruses deploy genomic accordions to adapt rapidly against host antiviral defenses. Cell 150, 831–841 22901812 10.1016/j.cell.2012.05.049 PMC3499626

[76] French A. R. Pingel J. T. Wagner M. Bubic I. Yang L. Kim S. Koszinowski U. Jonjic S. Yokoyama W. M. (2004) Escape of mutant double-stranded DNA virus from innate immune control. Immunity 20, 747–756 15189739 10.1016/j.immuni.2004.05.006

[77] Unterholzner L. Sumner R. P. Baran M. Ren H. Mansur D. S. Bourke N. M. Randow F. Smith G. L. Bowie A. G. (2011) Vaccinia virus protein C6 is a virulence factor that binds TBK-1 adaptor proteins and inhibits activation of IRF3 and IRF7. PLoS Pathog. 7, e1002247 21931555 10.1371/journal.ppat.1002247 PMC3169548

[78] Ferguson B. J. Benfield C. T. Ren H. Lee V. H. Frazer G. L. Strnadova P. Sumner R. P. Smith G. L. (2013) Vaccinia virus protein N2 is a nuclear IRF3 inhibitor that promotes virulence. J. Gen. Virol. 94, 2070–2081 23761407 10.1099/vir.0.054114-0 PMC3749055

[79] Kalverda A. P. Thompson G. S. Vogel A. Schröder M. Bowie A. G. Khan A. R. Homans S. W. (2009) Poxvirus K7 protein adopts a Bcl-2 fold: biochemical mapping of its interactions with human DEAD box RNA helicase DDX3. J. Mol. Biol. 385, 843–853 18845156 10.1016/j.jmb.2008.09.048

[80] Gerlic M. Faustin B. Postigo A. Yu E. C. Proell M. Gombosuren N. Krajewska M. Flynn R. Croft M. Way M. Satterthwait A. Liddington R. C. Salek-Ardakani S. Matsuzawa S. Reed J. C. (2013) Vaccinia virus F1L protein promotes virulence by inhibiting inflammasome activation. Proc. Natl. Acad. Sci. U.S.A. 110, 7808–7813 23603272 10.1073/pnas.1215995110 PMC3651467

[81] Postigo A. Cross J. R. Downward J. Way M. (2006) Interaction of F1L with the BH3 domain of Bak is responsible for inhibiting vaccinia-induced apoptosis. Cell Death Differ. 13, 1651–1662 16439990 10.1038/sj.cdd.4401853

[82] Wasilenko S. T. Stewart T. L. Meyers A. F. Barry M. (2003) Vaccinia virus encodes a previously uncharacterized mitochondrial-associated inhibitor of apoptosis. Proc. Natl. Acad. Sci. U.S.A. 100, 14345–14350 14610284 10.1073/pnas.2235583100 PMC283594

[83] Bowie A. Kiss-Toth E. Symons J. A. Smith G. L. Dower S. K. O'Neill L. A. (2000) A46R and A52R from vaccinia virus are antagonists of host IL-1 and Toll-like receptor signaling. Proc. Natl. Acad. Sci. U.S.A. 97, 10162–10167 10920188 10.1073/pnas.160027697 PMC27775

[84] Karplus P. A. Diederichs K. (2012) Linking crystallographic model and data quality. Science 336, 1030–1033 22628654 10.1126/science.1218231 PMC3457925

