# Supplementary material for: Vaccinia Virus Protein A49 Is an Unexpected Member of the B-cell Lymphoma (Bcl)-2 Protein Family
Source: J Biol Chem. 2015 Jan 20;290(10):5991–6002. doi: 10.1074/jbc.M114.624650 (PMC4358236; doi:10.1074/jbc.M114.624650)
Supplement: Supplemental Data [file supp_290_10_5991__index.html]

Vaccinia Virus Protein A49 is an Unexpected Member of the B-cell Lymphoma (Bcl)-2 Protein Family — Vaccinia Virus Protein A49 Is an Unexpected Member of the B-cell Lymphoma (Bcl)-2 Protein Family — Structure of Vaccinia Virus Protein A49 — Supplemental Data 

# Vaccinia Virus Protein A49 Is an Unexpected Member of the B-cell Lymphoma (Bcl)-2 Protein Family

## Supplemental Data

**Files in this Data Supplement:**

- Supplemental Material (.zip, 841 KB) - PDB files containing the conserved core of the Bcl-2 fold and of 35 representative Bcl-2 family proteins superposed on this core.
